# Hepatic thyroid signaling of heat-stressed late pregnant and early lactating cows

**DOI:** 10.1530/JOE-17-0066

**Published:** 2017-05-12

**Authors:** Joachim M Weitzel, Torsten Viergutz, Dirk Albrecht, Rupert Bruckmaier, Marion Schmicke, Armin Tuchscherer, Franziska Koch, Björn Kuhla

**Affiliations:** 1 Institute of Reproductive BiologyLeibniz Institute for Farm Animal Biology (FBN), Dummerstorf, Germany; 2 Institute of MicrobiologyErnst-Moritz-Arndt-University, Greifswald, Germany; 3 Veterinary PhysiologyVetsuisse Faculty, University of Bern, Bern, Switzerland; 4 Clinic for CattleEndocrinology Laboratory, University of Veterinary Medicine Hannover, Hannover, Germany; 5 Institute of Genetics and BiometryLeibniz Institute for Farm Animal Biology (FBN), Dummerstorf, Germany; 6 Institute of Nutritional Physiology ‘Oskar Kellner’Leibniz Institute for Farm Animal Biology (FBN), Dummerstorf, Germany

**Keywords:** thyroid hormone metabolism, thyroid-stimulating hormone, liver, dairy cow, heat stress

## Abstract

During the transition between late gestation and early lactation, dairy cows experience severe metabolic stress due to the high energy and nutrient requirements of the fetus and the mammary gland. Additional thermal stress that occurs with rising temperatures during the ongoing climate change has further adverse implications on energy intake, metabolism and welfare. The thyroid hormone (TH)-mediated cellular signaling has a pivotal role in regulation of body temperature, energy intake and metabolic adaptation to heat. To distinguish between energy intake and heat stress-related effects, Holstein cows were first kept at thermoneutrality at 15°C followed by exposure to heat stress (HS) at 28°C or pair-feeding (PF) at 15°C for 6 days, in late pregnancy and again in early lactation. Herein, we focused on hepatic metabolic changes associated with alterations in the hypothalamic–pituitary–thyroid axis in HS and PF animals. T_3_ and T_4_ levels dropped with HS or PF; however, in HS animals, this decline was more pronounced. Thyroid-stimulating hormone (TSH) levels remain unaffected, while plasma cholesterol concentrations were lower in HS than PF animals. Hepatic marker genes for TH action (*THRA*, *DIO1* and *PPARGC1*) decreased after HS and were lower compared to PF cows but only post-partum. Proteomics data revealed reduced hepatic amino acid catabolism ante-partum and a shift toward activated beta-oxidation and gluconeogenesis but declined oxidative stress defense post-partum. Thus, liver metabolism of HS and PF cows adapts differently to diminished energy intake both ante-partum and post-partum, and a different TH sensitivity is involved in the regulation of catabolic processes.

## Introduction

Thyroid hormone (TH) has a profound influence on normal development, differentiation and metabolism. Genomic actions of THs are mainly mediated and regulated by thyroid hormone receptors (THRs) (Cheng *et al.* 2010, Cioffi *et al.* 2013, Davis *et al.* 2013). THRs bind to TH response elements, which are located in promoter sequences of target genes but may also be positioned several thousand base pairs up- or downstream of the regulated gene (Cheng *et al.* 2010, Weitzel & Iwen 2011). The THRs belong to a group of transcription factors whose gene regulation function is depending on the presence or absence of their particular ligand (i.e. TH). Liganded and un-liganded THRs recruit cofactors which convert chromatin in an open or closed conformation, respectively (Astapova & Hollenberg 2013). Concentrations of thyroid hormones in the circulation are regulated via the negative feedback loop by the action of thyroid-stimulating hormone (TSH). Beside these regulatory mechanisms, the ligand itself can be modified e.g. via the action of deiodinases (Piehl *et al.* 2011, Gereben *et al.* 2015).

The combined action of THR together with its ligand regulates a wide variety of TH target genes via TH response elements. The primary THR targets include other transcription factors (e.g. the nuclear respiratory factor-1, *NRF1*) and transcriptional cofactors (e.g. PPARγ coactivator-1α, *PPARGC1*). These primarily regulated factors serve as intermediate factors, which regulate and orchestrate a second series of TH target genes (Weitzel & Iwen 2011). This combined action finally regulates numerous TH target genes among those who are responsible for mitochondrial activity and biogenesis (e.g. phosphoenolpyruvate carboxykinase (*PEPCK*), glycerol-3-phosphate dehydrogenase (*GPDH*) or mitochondrial ATP synthase subunit beta (*ATP5B*)).

TH is a profound regulator of body temperature. An excess of TH (hyperthyroidism) is associated with increased body temperature, whereas a TH deficiency (hypothyroidism) is associated with a decrease in body temperature (Mullur *et al.* 2014). Both statuses are accomplished by accelerated and dampened metabolic activities, respectively. Further, alterations in important thyroid hormone-triggered signal transduction pathways lead to modulations of body temperature. Transgenic mouse models carrying a targeted gene mutation in the *THRA* or the mitochondrial *GPDH* genes show impaired thermoregulation (Eto *et al.* 1999, Warner *et al.* 2013). Also, reciprocal mechanisms apply. Upon exposure to environmental heat, the affected organism responds with a decline in TH concentrations (Mullur *et al.* 2014).

In high-producing dairy cows, elevated ambient temperatures during the summer period induce metabolic heat stress, which has adverse effects on milk production, reproductive performance and animal welfare issues, among others. Counterregulatory mechanisms generally include an increased sweating rate, increased water intake and decreased feed intake, all contributing to reduce endogenous heat production (Lamp *et al.* 2015). However, the ratio of hepatic glucose output to milk lactose output is increased, whereas lipolysis and fat oxidation are not activated in heat-stressed lactating cows despite being in a catabolic state (Wheelock *et al.* 2010, Lamp *et al.* 2015). The shift in substrate utilization from fat to glucose has been proposed to diminish endogenous heat production while maintaining hepatic gluconeogenesis for fetus development in late gestation and milk production in early lactation (Koch *et al.* 2016). However, the molecular mechanisms underlying the shift in substrate utilization during heat stress have not been entirely elucidated so far. It seems likely that the TH system and TH-mediated signaling play a pivotal role in the control of substrate utilization and thus body temperature of heat-stressed cows. In order to distinguish between heat and energy intake-related effects, we analyzed the liver metabolism associated with the hypothalamic–pituitary–thyroid axis in late pregnant and early lactating Holstein cows kept either under heat stress (HS) or pair-fed (PF) conditions at thermoneutrality. We determined circulating endocrine hormones and the hepatic expression levels of known TH target genes as well as differentially regulated target genes in a holistic proteomics approach. The results of the study show that cows respond to HS by a more severe decline in plasma T_3_ and T_4_ but not TSH concentrations compared to PF cows and that early lactating but not late pregnant cows have reduced abundances of thyroid hormone receptor alpha (*THRA*), iodothyronine deiodinase 1 (*DIO1*) and *PPARGC1*, an activated hepatic beta-oxidation and gluconeogenesis and a concomitantly decline in oxidative stress defense enzymes.

## Methods

### Animals and sampling

Fourteen German Holstein dairy cows at the end of the 2nd parity were genotyped for HDP70.1 5′UTR 895 (Basirico *et al.* 2011) according to the genotype equally allocated to heat-stressed (HS, *n* = 7) or pair-feeding (PF, *n* = 7) group as described earlier (Lamp *et al.* 2015). The experiment was approved by the Ethic Committee of the State Government Mecklenburg-West Pomerania (Registration No. LALLF M-V/TSD/7221.3-1.1-074/12). All cows were not milked within the 7 weeks prior to the expected calving date. Animals received a total mixed ration twice daily at 07:00 h and 15:00 h. Both groups passed through a 13-day trial once in ante-partum (ap) (HSap and PFap) and post-partum (pp) stage (HSpp and PFpp).

The trial lasted from days 21 to 8 (±2.8) before and again from days 22 to 35 (±1.5) after parturition. Animals were halter trained and well adapted to climate chambers with a light cycle ranging from 06:00 to 19:00. The 13-day trial consisted of 6 days of period P1, one day of thermal transition (=day 1 of P2) and 6 further days of P2, each in the ap and pp period.

During period P1, both HS and PF groups were exposed to the same climate conditions (15°C, 63 ± 1% relative humidity (RH) resulting in a temperature-humidity-index (THI) of 60) with *ad libitum* feeding. On the following transition day, the air temperature was continuously increased to permanent 28°C for HS, but maintained at 15°C for PF animals (experimental period P2). RH equilibrated within 24 h to 52 ± 2% for HS cows resulting in a THI = 76. The THI was calculated according to the guidelines of the National Research Council (NRC 1971): THI = (1.8 × AT (°C) + 32) − (0.55 − 0.0055 × RH (%)) × (1.8 × AT (°C) − 26). Feed intake was recorded daily. Reduction of daily *ad libitum* intake of HS cows during P2 was calculated as percentage of the mean daily intake in P1 to provide the same amount of feed to PF cows in P2. Cows had free access to water, which was tempered to 28°C for HS and 15°C for PF cows during P2.

Cows were equipped with a jugular catheter (Certofix mono; B-Braun, Melsungen, Germany) to collect daily blood samples into 9 mL-monovettes (Sarstedt, Nümbrecht, Germany) containing EDTA before morning feeding. Blood samples were placed on ice and centrifuged immediately at 1570 ***g*** for 20 min at 4°C to obtain plasma, which was stored at −80°C before analysis.

### Plasma hormones and cholesterol

Total plasma triiodothyronine (T_3_) and thyroxine (T_4_) were measured by radioimmunoassay as described by Vicari and coworkers (2008). Plasma thyroid-stimulating hormone (TSH) concentrations were measured as described previously (Meyerholz *et al.* 2016) using an in-house ELISA. For the ELISA, an antibody targeted against bovine TSH (anti-bovine TSH, 1:10 pre-diluted, AFP-642482Rb) was obtained from the National Hormone and Peptide Program (NHPP, National Hormone and Peptide Program, NIDDK and Dr Parlow) and was diluted and used at a final dilution of 1:2500. The standard curve ranged from 0.2 to 100 ng/mL bovine TSH (AFP-8755B, obtained from the NHPP, NIDDK and Dr Parlow) that was dissolved in peptide buffer. The standards, controls and plasma samples (in triplicate) were added to a microtiter plate coated with the antibody, and the plate was then incubated for 24 h at RT. After washing the plate, biotin-labeled TSH (AFP-8755B, obtained from the NHPP, NIDDK and Dr Parlow) was added to all of the wells and incubated for 3 h. Then, a streptavidin horseradish peroxidase solution (Sigma Aldrich) was added, the substrate (containing tetramethylbenzidine, Sigma Aldrich) was pipetted after washing and the reaction was stopped after 15 min by adding sulfuric acid (2 M; Sigma Aldrich). The optical density was obtained at a wavelength of 450 nm, and the concentrations were calculated using Magellan software with the cubic spline modus (Magellan 3.11, Dortmund, Germany). The intra-assay CV was determined by measuring one bovine sample 20 times, and the result was 15.4%. The lowest detection limit of the ELISA was 0.6 ng/mL which was determined by using the last detectable concentration of a bovine serum sample that was serially diluted. Plasma cholesterol concentrations were analyzed photometrically (Abx Pentra 400, Horiba, Kyoto, Japan) using a cholesterol kit (No. 553-127, MTI Diagnostics, Idstein, Germany).

Liver biopsies were taken before morning feeding at the end of period P1 and on day 7 of P2, both ap and pp. To this end, the skin around the 12th intercostal space was anesthetized with 10 mL Isocaine 2% (Serumwerk, Bernburg, Germany), and a sample was taken with a tailor-made biopsy needle (Ø 6 mm). Biopsies were placed on absorbent paper to soak in blood, and the remaining liver tissues were snap frozen in liquid nitrogen and stored at −80°C until analysis. Due to severe sickness of individual cows, which were excluded from the trial, or due to limited tissue obtained in either P1 or P2, molecular analyses could only be performed on HSap *n* = 6, PFap *n* = 5, HSpp *n* = 6 and PFpp *n* = 4 cows.

### mRNA preparation and qRT-PCR

Tissues were mortared under liquid nitrogen, and total RNA was extracted from 50 mg tissue powder with TriFast Reagent (Peqlab, Erlangen, Germany). Concentration and quality of the extracted RNA were measured using a NanoDrop ND-1000 Spectrophotometer (Peqlab Biotechnologie GmbH). Ratios of absorbance at 260 and 280 nm of all preparations were about 2.0. RNA quality was further assessed using an Agilent 2100 Bioanalyzer, yielding RNA integrity numbers (RIN) of 9.1 ± 0.5. First-strand cDNA synthesis (750 ng total RNA) was completed using 2400 U RevertAid Reverse Transcriptase (Thermo Fisher Scientific) and 250 pmol random primers (Metabion International, Planegg/Steinkirchen, Germany). A negative control, without reverse transcriptase, was processed for each sample to detect possible contaminations of genomic DNA or environmental DNA. Aliquots (1 µL) of each RT reaction (1/20 of total) were primed, in each 10 µL PCR, using an iQ-SYBR green supermix (Bio-Rad Laboratories) and gene-specific oligonucleotides (final concentration of 0.2 µM). The sequences of specific bovine primers used are shown in Supplementary Table 2 (see section on supplementary data given at the end of this article). PCR was performed with 3 min at 94°C followed by 40 cycles of 10 s at 94°C; 30 s at 60°C; 225 s at 70°C, which were followed by a single elongation step of 7 min at 70°C using an iCycler (Bio-Rad Laboratories GmbH). The specificity of amplification was determined and confirmed by (i) melting curve analysis, (ii) agarose gel electrophoresis and (iii) by sequencing. Each PCR reaction was performed in duplicate, and the amount of transcripts was given as pg per 100 ng total RNA using standard curves for each transcript.

### Sample preparation and 2-DE analyses

Liver tissue powder (50 mg) was homogenized using a teflon pestle in 200 µL of 8 M urea, 50 mM Tris, 2% CHAPS, 40 mM DTT, 0.5% IPG buffer. After centrifugation (11,000 ***g***, 4°C, 20 min), the protein concentration in the supernatant was measured according to the Bradford method using bovine serum albumin (BSA) as standard. Where possible, individual samples were run in technical duplicates for the P1 and P2 period yielding 21 gels for 6 HSap, 19 gels for 5 PFap, 21 gels for 6 HSpp and 14 gels for 4 PFpp cows. A sample of 300 µg protein was added to 320 µL rehydration buffer (8 M urea, 2% CHAPS, 0.8% IPG-buffer, 18 mM DTT and a trace of bromophenol blue), mixed and loaded to 18 cm IPG (pH 3–10; Amersham) as previously described (Schaff *et al.* 2012). Active rehydration and IEF was performed at 50 V for 12 h followed by 500 V for 1 h, 1000 V for 1 h and 8000 V for 4 h 20 min using an Ettan IPGphor3 device (GE Healthcare). IPGs were equilibrated in buffer containing 50 mM Tris (pH 8.8), 30% glycerol, 6 M urea, 2% SDS, 1% DTT and then in the same buffer without DTT, but 2.5% iodoacetamide, each for 15 min. IPGs were transferred to 12.5% SDS-PAGE gels (20 × 20 × 0.1 cm) and embedded in low melting agarose. The gels were stained overnight in colloidal Rotiblue (Roth, Karlsruhe, Germany), de-stained 3 times in 15% methanol and 5% acetic acid and once in distilled water.

### Image analysis

Gels were scanned using an Epson Perfection 1250 scanner and saved as TIFF (8-bit gray scale). The 2-DE image analysis was carried out using Delta2D software version 4.6 (Decodon, Greifswald, Germany; http://www.decodon.com). The 40 gel images from the ap and the 35 gel images from the pp period were separately warped according to the ‘all to one’ warping strategy. A fusion image was created from all warped images containing all spots from all gels, each for the ap and pp period, respectively. After automatic spot detection on the ap and pp fusion images, spot boundaries were transferred to the original images and were quantified using the gray value of each spot to obtain the spot volume. Each spot volume was normalized to the total spot volume of each gel image (=100%) yielding the normalized spot volume in %. Only those spots were identified by mass spectrometry, which differed between the P1 and P2 period of HSpp by *P* < 0.1 while remaining unaffected in PFpp cows. Spots differing between the P1 and P2 period of PF cows were not considered. Identified spots are displayed on representative 2D gels in Supplementary Fig. 1.

### Mass spectrometry

Protein identification was performed by a refined method described and compiled previously (Schaff *et al.* 2012). Briefly, protein spots were punched out using an Ettan spot cutter (Amersham) with a Ø 2 mm picker head. Spots were transferred into 96-well plates, tryptic digested and subsequently spotted on a MALDI-target. The molecular masses of tryptic digests were measured on a 5800 MALDI TOF/TOF Analyzer (Applied Biosystems). The spectra were recorded in a mass range from 900 to 3700 Da with a focus on 1600 Da. For the record of one main spectrum, 30 measuring points per tryptic digest spot were exposed to laser bombardment. At each measuring point, 200 laser shots were set generating 200 sub-spectra, which were subsequently accumulated to one main spectrum. When the autolytical fragments of trypsin with (M+H)+ *m*/*z* at 1045.556 and 2211.104 reached a S/N of at least 40, an internal calibration was automatically performed as two-point calibration. The standard mass deviation was less than 0.15 Da. After calibration, the peak lists were created by using the ‘peak to mascot’ script of the 4000 Series Explorer Software (version 3.5). Selected settings were the following: mass range from 900 to 3800 Da, peak density of 15 peaks per 200 Da, minimal area of 100 and maximal 60 peaks per spot. The peak list was created for an S/N of 10.

To confirm the results obtained by MALDI-TOF-MS, MALDI-TOF-TOF analysis was performed on the 5800 MALDI TOF/TOF Analyzer (Applied Biosystems). The three strongest peaks of the TOF-spectra were selected automatically and measured. For one main spectrum 25 sub-spectra with 255 shots per sub-spectrum were accumulated using a random search pattern. The internal calibration was automatically performed as one-point calibration with (M+H)+ *m*/*z* at 175.119 or with Lys (M+H)+ *m*/*z* at 147.107 reached a S/N of at least 5. The peak lists were created for a S/N of 7. Selected settings were the following: mass range from 60 Da to (precursor–20) Da, peak density of 15 peaks per 200 Da, minimal area of 100 and maximal 65 peaks per precursor.

For the identification of proteins, a data base search with peptide mass fingerprint (PMF) was performed against NCBInr (National Center for Biotechnology Information, http://www.ncbi.nlm.nih.gov/) database using the Mascot search engine version 2.4.1 (Matrix Science, London, UK). Search parameters were the following: taxonomy: ‘all entries’; variable modifications: ‘carbamidomethyl (C)’ and ‘oxidation (M)’; precursor tolerance ‘±50 ppm’; peptide charge ‘1+’; MS/MS fragment tolerance ‘0.5 Da’; MS/MS tolerance ‘0.5 Da’; ‘monoisotopic’. Mascot criteria for acceptance of protein identifications by PMF were ‘number of masses matched’, ‘number of masses not matched’. A Mascot overall Protein Score greater than 75 fulfilled the 95% confidence interval (C.I. %; *P* < 0.05) and are listed in Supplementary Table 1.

### Western blot

Liver tissue (30 mg) was homogenized in lysis buffer containing 50 mM Tris–HCl, 1 mM EDTA, 10 mM NaF, 1% IGPEAL CA-630, 0.1% Triton X100, 0.5% deoxycholic acid (DCA) and 0.1% SDS (pH 7.8). Protein concentrations were determined using the Bradford method. Equal amounts of protein (25 µg) were separated by SDS-PAGE. Separated proteins were transferred to a nitrocellulose membrane, stained with Ponceau S, scanned and saved as image file. Membranes were blocked with 3% BSA in Tris-buffered saline (TBS, pH 7.8) containing 0.1% Tween 20 (TBST) for 1 h and then incubated overnight with the following primary antibodies against: ATP synthase subunit beta (ATP5B; sc-166338, Santa Cruz Biotechnology), protein disulfide-isomerase precursor (P4HB; AV48151, Sigma-Aldrich), regucalcin (RGN; 17947-1-AP; Protein Tech Group). After incubation, membranes were washed in TBST, incubated for 1 h at room temperature with the corresponding secondary antibody (goat anti-rabbit IgG HRP, sc-2004, Santa Cruz Biotechnology) and washed again in TBST. Chemiluminescent reagents were applied, and blots were exposed to hyperfilms (GE Healthcare). Hyperfilms were scanned, and bands were quantified using ImageJ (version 1.49). Optical density of immunostained bands was normalized to corresponding Ponceau S-stained lanes.

### Statistical analysis

Due to interindividual variation between HS and PF cows in the P1 period, plasma concentrations of total T_3_, total T_4_, TSH and cholesterol were baseline corrected to the mean value of the last three days of thermoneutral P1 conditions. Plasma T_3_, T_4_ and TSH data were analyzed by repeated measurement ANOVA with the MIXED procedure of SAS/STAT software (Version 9.4, SAS Institute Inc., Cary, NC, USA). The ANOVA model contained the fixed factors group (levels: HS, PF), time (challenge day) and the interaction group × time. Repeated measures on the same animal were taken into account by the REPEATED statement of the MIXED procedure. The block diagonal residual covariance matrix was unstructured for baseline-corrected values (T_3_, T_4_, TSH and cholesterol), whereas it was autoregressive for plasma concentrations of total T_3_ and total T_4_. Least-squares means (LSM) and their standard errors (s.e.) were computed for each fixed effect in the models, and all pairwise differences of LSM were tested by the Tukey–Kramer procedure. The SLICE statement of the MIXED procedure was used for performing partitioned analyses of the LSM for the interaction group × time. For PCR and proteome analyses, differences between periods P1 and P2 in the same group were analyzed using the Wilcoxon signed rank sum test included in the UNIVARIATE procedure of Base SAS. Analysis of differences between two groups of the same period and the same productive stage was performed using the exact Wilcoxon–Mann–Whitney test of SAS/STAT. Data are given as mean ± s.e. Results were considered as statistical significant at *P* < 0.05.

## Results

### Thyroid hormones and TSH

**Figure 1:**
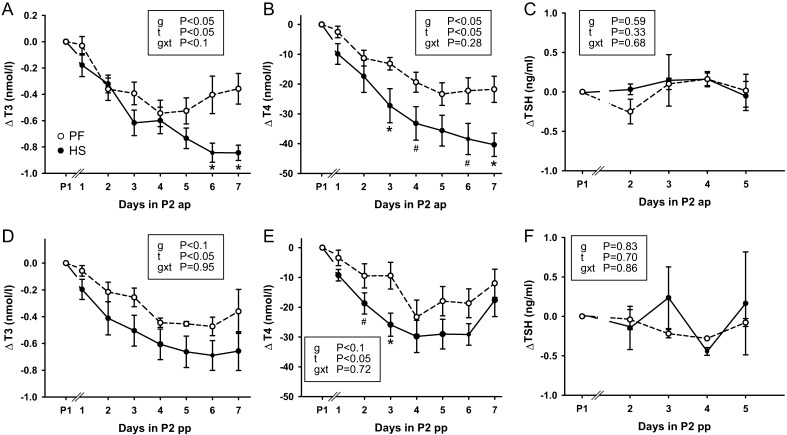
Baseline-corrected plasma concentrations of total T_3_, total T_4_ and TSH determined after *ad libitum* feeding at thermoneutrality in P1, the transition day (day 1 of P2) and during the 6 subsequent challenge days of period P2 of either heat stress (HS, filled circles) or pair-feeding at thermoneutrality (PF, open circles) in the ante-partum (ap) and post-partum (pp) period, respectively. Data are given as mean ± s.e.m. *P* values for the effects between groups (g), over time (t) and their interaction (g × t) are presented within graphs. * indicates *P* < 0.05 and ^#^*P* < 0.1 for pairwise comparisons between groups. HSap *n* = 7, PFap *n* = 6, HSpp *n* = 6, PFpp *n* = 6.

After increasing ambient temperature to 28°C (THI = 76), HS dairy cows responded with declining total T_3_ and total T_4_ concentrations both in the ap (Fig. 1A and B) and pp period (Fig. 1D and E). The T_3_ level dropped from ~2 nmol/L at thermoneutrality (15°C, THI = 60) to ~1.2 nmol/L after challenge (day 7 in P2), whereas T_4_ levels dropped from ~65 nmol/L at thermoneutrality to ~35 nmol/L in ap animals (*P*_time_ < 0.001; Supplementary Fig. 2). The T_3_/T_4_ ratio remained unaffected during treatments (data not shown). Total plasma T_3_ was ~0.3 nmol/L, and total plasma T_4_ was ~22 nmol/L lower in HSpp than HSap animals, both before (P1) and after challenge, while the extent of reduction over time (*P*_time_ < 0.05) was comparable in both periods (Fig. 1B and E). Also, pair-fed animals responded by a reduction of TH levels both in the ap and pp period. However, plasma T_3_ and T_4_ concentrations declined or tended to decline lesser in PFap compared to HSap cows (T_3_: *P* < 0.05 for days 6 and 7 of P2; T_4_: *P* < 0.1 for days 4 and 6 of P2, *P* < 0.05 for days 3 and 7 of P2; Tukey–Kramer). TSH concentrations remained constant at approximately 1 ng/mL with no significant alterations over time or between animals, and independent of whether animals were in the ap or pp period (Fig. 1C and F). In the pp period, total T_3_ and T_4_ plasma concentrations tended to be lower in HS than PF cows (*P*_group_ < 0.1); however, pairwise comparisons revealed no differences after the 7-day challenge period (Fig. 1D and E). Only on days 2 and 3 of P2, total T_4_ differed between PFpp and HSpp cows (Fig. 1E).

### Hepatic intracellular thyroid signaling

**Figure 2:**
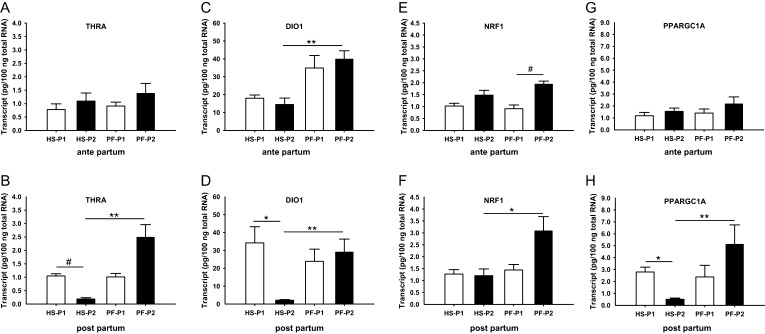
mRNA abundances of (A, B) thyroid hormone receptor alpha (*THRA*), (C, D) deiodinase 1 (*DIO1*), (E, F) nuclear respiratory factor 1 (*NRF1*), and (G, H) peroxisome proliferator-activated receptor gamma coactivator 1-alpha (*PPARGC1A*) in liver samples obtained after *ad libitum* feeding at thermoneutrality (P1; open bars) and after heat stress (HS; filled bars) or pair-feeding at thermoneutrality (PF; filled bars) at day 7 in P2, both in the ante-partum and post-partum period, respectively. ** indicates *P* < 0.01, *P < 0.05, and ^#^*P* < 0.1). Data are from HSap *n* = 6, PFap *n* = 5, HSpp *n* = 6, PFpp *n* = 4 and are presented as mean ± s.e.m.

To examine whether the different or the tending different declines in circulating TH concentrations in HS and PF animals affect cellular thyroid signaling, we measured various hepatic marker genes for T_3_ action. In the ap period, exposure to HS or isocaloric feeding at thermoneutrality did not significantly alter RNA concentrations of *THRA*, *DIO1*, *NRF1* and *PPARGC1* from P1 to P2 (Fig. 2A, C, E and G). Only the *NRF1* RNA abundance tended to increase in PF cows relative to *ad libitum* feeding at thermoneutrality (Fig. 2E). In addition, *DIO1* mRNA abundance differed significantly between PF and HS cows in the P2 state; however, this difference rather arise from the different expression levels in P1 (Fig. 2C). In the pp period, however, transition from thermoneutrality to HS but not to PF reduced gene expression levels for *THRA*, *DIO1* and *PPARGC1A* (*P* < 0.05) by 82–94% (Fig. 2B, D and H), However, mRNA abundance of *NRF1* was not affected by HS, yet, abundances of *NRF1*, *THRA*, *DIO1* and *PPARGC1A* were 2.8–14 times greater in PF compared to HS-challenged animals (Fig. 2B, D, F and H).

### Hepatic metabolism

To elucidate hepatic metabolic processes potentially associated with the different plasma TH concentrations between HSap and PFap cows and different cellular signal activation between HSpp and PFpp cows, we performed a 2D-GE based proteome approach. Out of 1365 spots detected at the ap-fused image and out of the 1102 spots detected at the pp fused image, we picked only those spots whose expression differed between P1 and P2 by *P* < 0.1 in HS but not in PF animals.

Out of 114 spots differentially expressed between P1 and P2 in HSap animals, and out of 82 spots differentially expressed between P1 and P2 in HSpp animals, we identified 18 spots for the ap and 20 spots for the pp period by mass spectrometry and data base search (Table 1). In the ap period, five enzymes involved in amino acid degradation, namely isovaleryl-CoA dehydrogenase (IVD), 3-hydroxyisobutyrate dehydrogenase (HIBADH), beta-ureidopropionase (UPB1), arginase-1 (ARG1) and glycine amidinotransferase (AGAT) were found to be 20–38% lower expressed after heat challenge (Table 1). The proteasome subunit alpha (PSMA2), which is involved in protein degradation, coproporphyrinogen-III oxidase (CPOX), which is involved in haem biosynthesis, as well as ferritin light chain (FTL), the major iron storage protein, were all 1.4–2.7 fold upregulated, whereas 4-trimethylaminobutyraldehyde dehydrogenase (ALDH9A1), which is involved in carnitine biosynthesis, was 1.2-fold downregulated (Table 1). Among enzymes involved in carbohydrate and energy metabolism, UTP-glucose-1-phosphate uridylyltransferase (UGP1) was 1.3-fold upregulated, whereas galactokinase (GALK1), NADH-ubiquinone oxidoreductase (complex I), adenosine kinase (ADK) and carbonyl reductase 1 (CBR1) were 1.2–1.5-fold downregulated (Table 1). Furthermore, three spots coding for catalase (CAT) were all found 1.2–1.4 fold lower expressed after heat exposure (Table 1).

**Table 1:** Quantity of liver protein spots differentially expressed between the P1 and P2 period of ante-partum and post-partum heat-stressed but not pair-fed cows.

Stage	**Gel spot No.**	Description	Gene name	**Mean rel. abundance in P1** (Vol%)	**Mean rel. abundance in P2** (Vol%)	**Ratio P2/P1**	P value
Ante-partum	588	Arginase-1	ARG1	0.094	0.065	0.69	0.031
	524	Isovaleryl-CoA dehydrogenase	IVD	0.039	0.025	0.62	0.094
	782	3-Hydroxyisobutyrate dehydrogenase	HIBADH	0.061	0.048	0.79	0.031
	492	Glycine amidinotransferase	AGAT	0.303	0.243	0.80	0.031
	528	Beta-ureidopropionase	UPB1	0.087	0.063	0.72	0.031
	786	Proteasome subunit alpha	PSMA2	0.008	0.021	2.67	0.031
	628	Coproporphyrinogen-III oxidase	CPOX	0.015	0.029	1.99	0.063
	995	Ferritin light chain	FTL	0.038	0.054	1.41	0.031
	1339	4-Trimethylamino-butyraldehyde dehydrogenase	ALDH9A1	0.160	0.134	0.84	0.063
	423	UTP-glucose-1-phosphate uridylyltransferase	UGP1	0.200	0.262	1.31	0.031
	639	Galactokinase	GALK1	0.102	0.078	0.77	0.063
	736	Galactokinase	GALK1	0.046	0.036	0.78	0.063
	241	NADH-ubiquinone oxidoreductase, complex 1	–	0.029	0.020	0.67	0.063
	1345	Adenosine kinase	ADK	0.207	0.179	0.86	0.031
	768	Carbonyl reductase 1	CBR1	0.100	0.081	0.81	0.063
	377	Catalase	CAT	0.593	0.480	0.81	0.063
	1350	Catalase	CAT	0.300	0.234	0.78	0.031
	574	Catalase	CAT	0.288	0.203	0.70	0.031
Post-partum	199	3-Ketoacyl-CoA thiolase	ACAA2	0.202	0.311	1.54	0.063
	207	3-Ketoacyl-CoA thiolase	ACAA2	0.058	0.114	1.21	0.063
	327	3-Ketoacyl-CoA thiolase	ACAA2	0.081	0.121	1.51	0.063
	1004	Acetyl-coenzyme A acetyltransferase 2	ACAT2	0.125	0.092	0.73	0.063
	1005	Acetyl-coenzyme A acetyltransferase 2	ACAT2	0.108	0.063	0.58	0.063
	1022	Glycerol-3-phosphate dehydrogenase	GPDH	0.109	0.075	0.68	0.063
	183	Serine hydroxymethyltransferase	SHMT	0.059	0.120	1.26	0.063
	170	UTP-glucose-1-phosphate uridylyltransferase	UGP1	0.269	0.433	1.61	0.063
	191	Pyruvate carboxylase	PC	0.002	0.020	1.32	0.063
	194	Fumarate hydratase	FH	0.054	0.083	1.55	0.063
	315	Carbonyl reductase 1	CBR1	0.318	0.631	1.98	0.063
	862	Regucalcin isoform X1	REG1	1.137	0.910	0.80	0.063
	893	Sorbitol dehydrogenase	SDH	0.235	0.190	0.81	0.063
	663	Profilin 1	PFN1	0.039	0.037	0.96	0.063
	937	Protein disulfide-isomerase A3	PDIA3	0.331	0.218	0.66	0.063
	380	Peroxiredoxin 6	PRDX6	0.079	0.056	0.71	0.063
	400	Thioredoxin-dependent peroxide reductase	PRDX3	0.181	0.077	0.42	0.063
	890	Thiosulfate sulfurtransferase	TST	0.249	0.194	0.78	0.063
	934	Catalase	CAT	0.028	0.021	0.75	0.063
	1066	Persulfide dioxygenase	ETHE1	0.062	0.030	0.48	0.063

In the pp period, we found the pyridoxal phosphate-dependent enzyme serine hydroxymethyltransferase (SHMT) and three spots coding for 3-ketoacyl-CoA thiolase (ACAA2) 1.2–1.5 fold greater, but two acetyl-CoA acetyltransferase (ACAT2) spots and one *GPDH* spot 1.3–1.7-fold lower expressed after HS challenge (Table 1). Enzymes involved in glucose and energy metabolism were UGP1, pyruvate carboxylase (PC), fumarate hydratase (FH) and CBR1 which were 1.3–2-fold upregulated, while regucalcin 1 (RGN1) and sorbitol dehydrogenase (SDH) were 1.2–1.5-fold downregulated (Table 1). Profilin 1 (PFN1), which regulates actin polymerization, protein disulfide-isomerase A3 (PDIA3), but also enzymes involved in the xenobiotic and oxidative stress defense machinery, namely thiosulfate sulfurtransferase (TST), thioredoxin-dependent peroxide reductase (PRDX3), peroxiredoxin 6 (PRDX6), CAT and persulfide dioxygenase (ETHE1) were downregulated after HS challenge (Table 1).

**Figure 3:**
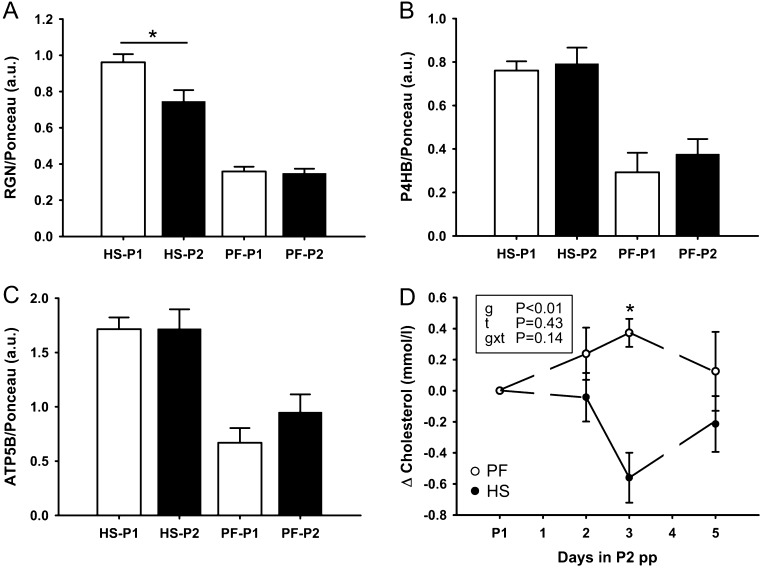
Western blot analysis of regucalcin (RGN) (A), protein disulfide-isomerase precursor (P4HB) (B) and ATP synthase subunit beta (ATP5B) (C), relative to Ponceau staining. Liver samples were obtained after *ad libitum* feeding at thermoneutrality (P1; open bars) and after heat stress (HS; filled bars) or pair-feeding at thermoneutrality (PF; filled bars) at day 7 in P2 of the post-partum period. Baseline-corrected plasma cholesterol concentrations obtained after *ad libitum* feeding in P1 and during HS (filled circles) or PF (open circles) in P2 of the post-partum period (D). Data are from HSpp *n* = 6, PFpp *n* = 4 and are presented as mean ± s.e.m. * indicates *P* < 0.05.

Next, we examined whether changes in regucalcin (spot 862) and protein disulfide-isomerase (spot 937) abundances can be verified by Western blot analysis. After heat exposure, regucalcin abundance was found significantly lower than in the preceding period P1 (Fig. 3A). By using an antibody directed against protein disulfide-isomerase precursor protein (P4HB) (Fig. 3B), we could not confirm changes in PDIA3 abundance as identified by the proteome approach (Table 1). Also, the secondary T_3_ marker gene ATP5B was not regulated on the protein level in response to heat stress or pair-feeding in the pp period (Fig. 3C). Further, as ACAT2 is known to be involved in many metabolic pathways, among them in hepatic acetoacetyl-CoA and cholesterol synthesis, we examined whether the decrease in ACAT2 in response to HS would be associated with declining plasma cholesterol concentrations. Exposure to HS leads to significantly lower plasma cholesterol concentrations compared to PF on day 3 of challenge but not on day 7 of the pp challenge (Fig. 3D).

## Discussion

Food restriction in dairy cows either in the ap or pp stage leads to a decrease in triiodothyronine and thyroxine concentrations by 40–47% after a 7 -day challenge. This has been expected as essential in order to ensure the demand for a reduced metabolic rate and to confine increase in body temperature in these animals (McGuire *et al.* 1991, West 2003, Lamp *et al.* 2015). Heat-stressed animals kept at 28°C (THI = 76) respond with an even more pronounced decline in T_3_ and T_4_ levels (Fig. 1), and the extent of reduction was more prominent in HS compared to PF animals in the ap state and tended to be different for total plasma T_4_ concentrations in the pp period. This again is in agreement to previous data observed in beef cattle as well as in lactating cows (Magdub *et al.* 1982, Al-Haidary *et al.* 2001). However, the major contributor of declining TH concentrations is the reduction in feed intake, while HS adds only a minor portion to the reduction. TSH levels remain constant over the observation period both in the PF as well as in the HS group (Fig. 1C and F). This is contradicting to a previous report by Kahl *et al*. (2015) in which the authors describe reduced TSH levels in heat-stressed steers. Differences in the experimental setting, between breeds, gender and physiological status may account for these diverse observations. More specifically, in the study by Kahl *et al*. (Kahl *et al.* 2015) male animals have been observed, whereas we herein investigated female animals three weeks before or three weeks after parturition. Further, heat stress has been induced in a daily cycling manner, whereas we used a constant temperature regime. A constant TSH level, as observed in our study, indicates that TH levels are tailored to meet the requirement in our experimental design over the observation period.

In a next step, we investigated hepatic marker genes which are essential for thyroid hormone-dependent gene regulation in liver. After binding of TH to THR these liganded nuclear receptors regulate a series of primary TH target genes via direct binding to TH response elements in the promoter region of these target genes (‘early’ gene regulation). The un-liganded THR regulates gene transcription in an opposite manner, and additionally many non-genomic actions of TH have been described which further fine tune the action of the hormone in different tissues (Cheng *et al.* 2010, Astapova & Hollenberg 2013, Cioffi *et al.* 2013, Davis *et al.* 2013). Among the primary THR target genes, the transcription factor *NRF1*, the transcriptional coactivator *PPARGC1* and the TH converting enzyme *DIO1* have been described to be directly regulated via a TH response element (Jakobs *et al.* 1997, Wulf *et al.* 2008). Both, *NRF1* and *PPARGC1* genes are cooperatively involved in regulating mitochondrial biogenesis in the liver (Weitzel & Iwen 2011), whereas the main activity of DIO1 is to convert T_4_ into the biologically more potent T_3_ (Gereben *et al.* 2015). All these target genes are positively regulated upon T_3_ stimulus and regulate specific key steps in TH action in the liver. Interestingly, these marker genes responded differentially in the ap and pp period (Fig. 2). In the ap state, the abundance of marker genes did not change when cows were transferred from thermoneutrality to heat and only *DIO1* differed between HS and PF cows, although total T_3_ and T_4_ plasma concentrations declined even more in HS than PF animals. By contrast, in the pp state, we found significantly reduced *THRA*, *DIO1* and *PPARGC1A* transcript levels, both by comparing HS to *ad libitum* fed (HS-P1) and by comparing HS-P2 to PF-P2 cows, but these changes were not associated with different T_3_ and T_4_ plasma concentrations, at least not on day 7 of challenge. These results indicate that TH-mediated signaling in early lactation but not in late pregnancy is specifically regulated due to increased temperature but not due to accompanied reduced energy intake. Our results further demonstrate diminished hepatic TH sensitivity in HS late pregnant cows. The non-responsiveness of TH-mediated signaling in HS late pregnant cows may be due to the level of total plasma T_4_, which was generally greater in the ap compared to the pp period, both in the basal state P1 as well as after challenge. However, the post-partum mitochondrial activity regulated by the transcription factor–coactivator complexes *THRA/PPARGC1A* and/or *NRF1/PPARGC1A* during HS seems to be dampened and might account for the switch from fat to glucose utilization as observed previously (Eslamizad *et al.* 2015). Interestingly, a decline of DIO1 enzyme activity has also been described in the above-mentioned study by Kahl and coworkers (Kahl *et al.* 2015). By contrast, we did not find a significant alteration in steady-state protein concentrations of ATP5B (Fig. 3C) as well as not for protein disulfide-isomerase precursor (P4HB) (Fig. 3B). Although ATP5B is known as a secondary TH target, a direct regulation via a TH response element has not been described for both genes. Indeed, hormone-mediated alterations of ATP5B and P4HB gene transcription have been described with a delay of 72 h which is followed by an alteration of protein concentrations earliest detectable ~4–5 days after TH stimulus (Obata *et al.* 1988, Weitzel & Iwen 2011). Possibly, the treatment time of only 7 days (including one day of thermal transition) in our experimental setting might be too short in order to induce manifest expression alterations. In addition, in post-partum PF cows, the abundance of *THRA*, *NRF1*, *DIO1* and *PPARGC1A* was greater than in HS cows, a regulation which might help to switch hepatic metabolism toward fatty acid oxidation during times of energy deficiency at thermoneutrality.

A switch of metabolic rate and a switch in substrate oxidation from thermoneutrality to heat exposure as discussed above and described earlier (Lamp *et al.* 2015) are also evident from our proteomics approach. Of note, we only analyzed those protein spots which were (i) differentially expressed between P1 and P2 of HS cows while (ii) remaining unaffected in PF cows. Thus, differential protein abundances listed in Table 1 can be specifically attributed to heat stress but not to reduced energy intake.

Several enzymes related to fat metabolism were found differentially expressed after heat exposure in the pp but not ap period. The ACAA2 protein was upregulated as compared to thermoneutral conditions, suggesting activated hepatic beta-oxidation. This finding seems to contradict earlier results demonstrating that lactating dairy cows are refractory to lipolysis and accordingly do not increase whole-body fat oxidation when adapting to ambient heat (Lamp *et al.* 2015), but the latter does not exclude that fatty acid oxidation may be activated in other organs. On the mRNA transcript level, hepatic mitochondrial beta-oxidation enzymes were not affected when cows in early lactation were heat stressed (Koch *et al.* 2016). As gene transcription costs up to 10% of the total cellular energy, oligonucleotide synthesis is often suppressed to confine endogenous heat production (Storey 2015). Thus, other mechanisms, primarily epigenetic and post-translational protein modifications gain importance in the control of enzyme activities during hypometabolism (Storey 2015), but whether the protein spots identified as ACAA2 were post-translationally modified need to be determined in future studies. Upregulation of UGP1, an enzyme involved in glycosylation of proteins, points to activated post-translation both in the ap and pp stage, which supports our assumption above. However, increased hepatic fatty acid oxidation during pp heat stress should generate substantial amounts of NADH, which in turn serve as substrate for an activated gluconeogenic pathway (see below). A further link between carbohydrate and lipid metabolism is controlled by GPDH. GPDH catalyzes the reversible reduction of dihydroxyacetone phosphate into glycerol 3-phosphate and finally glycerol and serves as a regulator controlling the provision of electrons to the electron transport chain in the mitochondria. Thus, reduced abundance of GPDH during pp heat stress indicates reduced hepatic lipid biosynthesis and a reduced electron flux through the respiratory chain, thereby reducing endogenous heat production while maintaining electron provision for gluconeogenesis. In addition, ACAT2, an enzyme involved in ketone body and cholesterol production, was lower expressed after the pp heat stress challenge, explaining sustained plasma beta-hydroxybutyrate concentrations in HS while they increased in PF early lactating cows (Koch *et al.* 2016). Further, downregulation of ACAT2 agrees to the declining plasma cholesterol concentrations of HS cows post-partum.

The hepatic amino acid catabolism was found differently regulated in the ap and pp period. While the amino acid catabolism is reduced after heat stress in late pregnant cows, it is activated in heat-stressed early lactating cows. The reduced expression of IVD, HIBADH, UPB1, and ARG1 in heat-stressed late pregnant cows together with increased expression of PSMA2 indicates intensified hepatic protein degradation but diminished branched-chain amino acids catabolism, likely because to direct these amino acids to the rapidly growing fetus. These data agree to a recent finding demonstrating that plasma urea concentrations do not increase after heat stress of pregnant cows (Koch *et al.* 2016). As a consequence of the reduced amino acid catabolism, mitochondrial energy production should also be reduced and accordingly complex I, ADK1 and CBR1 were lower expressed in late pregnant cows after heat exposure. On the contrary, upregulation of SHMT and ACAT2 proteins post-partum points to a stimulated hepatic amino acid catabolism aligned with significantly increased plasma urea concentrations described for HS but not PF lactating cows (Koch *et al.* 2016), probably because there is no need any more to transfer amino acids to the fetus. Instead, amino acids can be catabolized to produce energy leading inevitably to an increased urea cycling. Although we did not observe upregulation of urea cycle enzymes either at protein or at transcriptional level (Koch *et al.* 2016), the carbon chain of amino acids entered the TCA cycle and accordingly we found increased abundance of FH protein after heat exposure. Fumarate is converted to malate and the latter further to oxaloacetate, the main precursor for gluconeogenesis. In the present study, we found increased abundance of PC protein during pp heat stress, suggesting activated gluconeogenesis. In line with this, Shahzad *et al.* (Shahzad *et al.* 2015) reported a greater *PC* mRNA abundance in transition cows with summer compared to winter calving, although this might be due to the lower feed intake as well. However, lactating cows adapt to heat by increasing carbohydrate utilization (Rhoads *et al.* 2009, Lamp *et al.* 2015), and increased gluconeogenesis may meet these increased glucose requirements.

It has been shown that administration of T_3_ results in transcriptional upregulation of enzymes with oxidative stress defense characteristics such as superoxide dismutase (*SOD*) and *CAT* (Weitzel *et al.* 2001, 2003). Vice versa, reduction in plasma T_3_ concentration in HSap animals is accompanied by a reduction of the stress defense enzyme CAT, and thus corresponds to the declined hepatic mRNA abundance of *CAT* after heat exposure of early lactating dairy cows (Koch *et al.* 2016). Moreover, the trend in declining plasma T_3_ concentration in HSpp animals is associated by reduction of the stress defense enzymes TST, PRDX3, PRDX6, ETHE1 and also CAT, strongly suggesting the involvement of T_3_- and THRA-mediated signaling in this adaptation process. Downregulation of these defense enzymes seems to be a mechanism to allow for accumulating levels of peroxides and other reactive oxygen species, which in turn may activate transcription factors such as heat shock factor 1 (*HSF1*) or hypoxia-inducible factor 1 (*HIF-1*), both known to trigger cellular heat stress responses (Klumpen *et al.* 2017).

Taken together, our data indicate that Holstein cows responded with a decline in T_3_ and T_4_ to ambient heat or pair-feeding, but the extent of the decline was greater under heat-stressed conditions, particularly in the ap and as a trend in the pp period. However, absolute plasma T_4_ concentrations reached lower levels pp than ap. Hepatic marker genes of TH action did not respond in late pregnant cows indicating reduced TH sensitivity, likely to confine amino acid catabolism in order to direct amino acids to the fetus. On the other hand, hepatic mRNA abundances of *THRA*, *DIO1*, *NRF1* and *PPARGC1* were lower in HS than PF challenged early lactating cows, thus allowing TH-signal transduction to facilitate amino and fatty acid catabolism as well as gluconeogenesis during heat stress. However, activation of these metabolic pathways during heat stress is accompanied by diminished oxidative defense in early lactating cows.

## Supplementary Material

Supporting Figure 1

Supporting Figure 2

Supporting Table 1

Supporting Table 2

## Declaration of interest

The authors declare that there is no conflict of interest that could be perceived as prejudicing the impartiality of the research reported.

## Funding

This study was supported by the core budged of the Leibniz Institute for Farm Animal Biology (FBN). This research did not receive any grant from the commercial sector.
