# Supplementary figures and images for: Hepatic thyroid signaling of heat-stressed late pregnant and early lactating cows
Source: J Endocrinol. 2017 May 12;234(2):129–41. doi: 10.1530/JOE-17-0066 (PMC5516449; doi:10.1530/JOE-17-0066)

Supplementary Figure 1

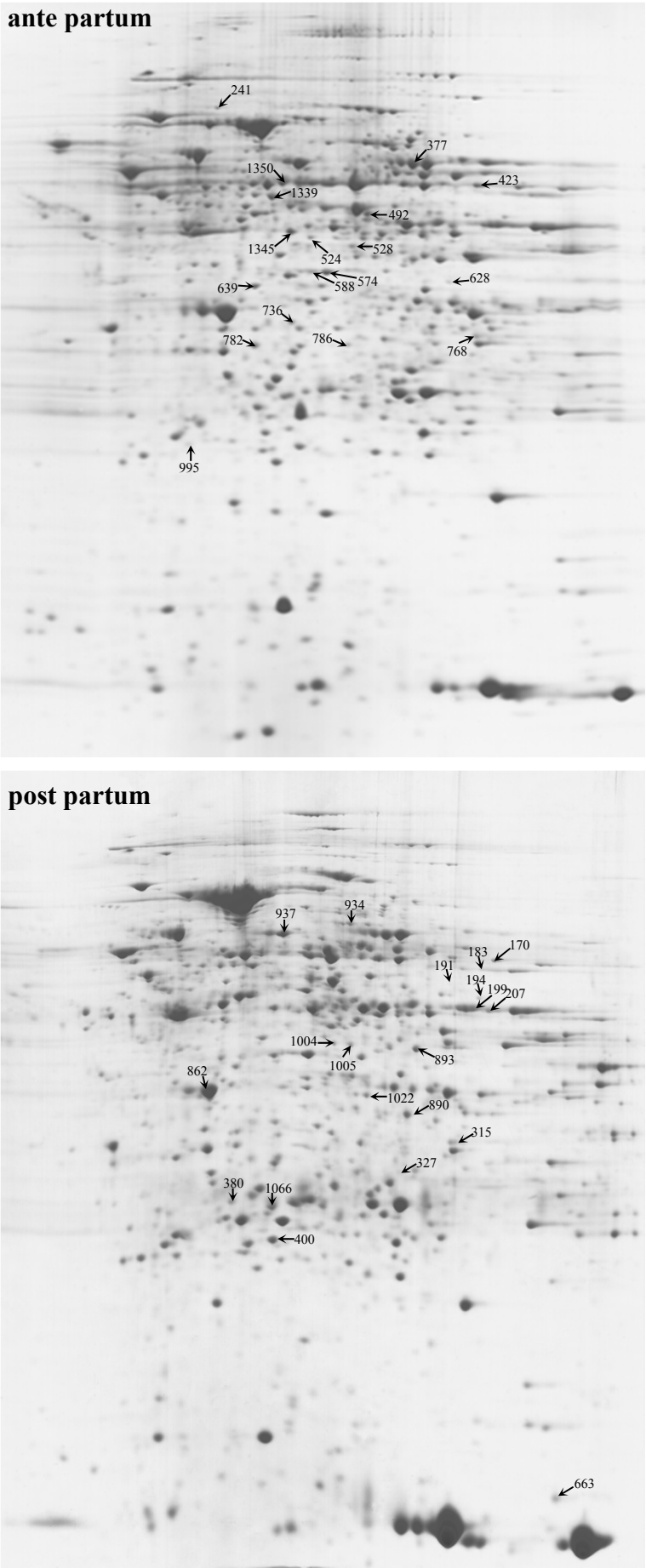

Supplement: Supporting Figure 1 [file joe-234-129-s001.pdf]

## Supplementary Figure 2

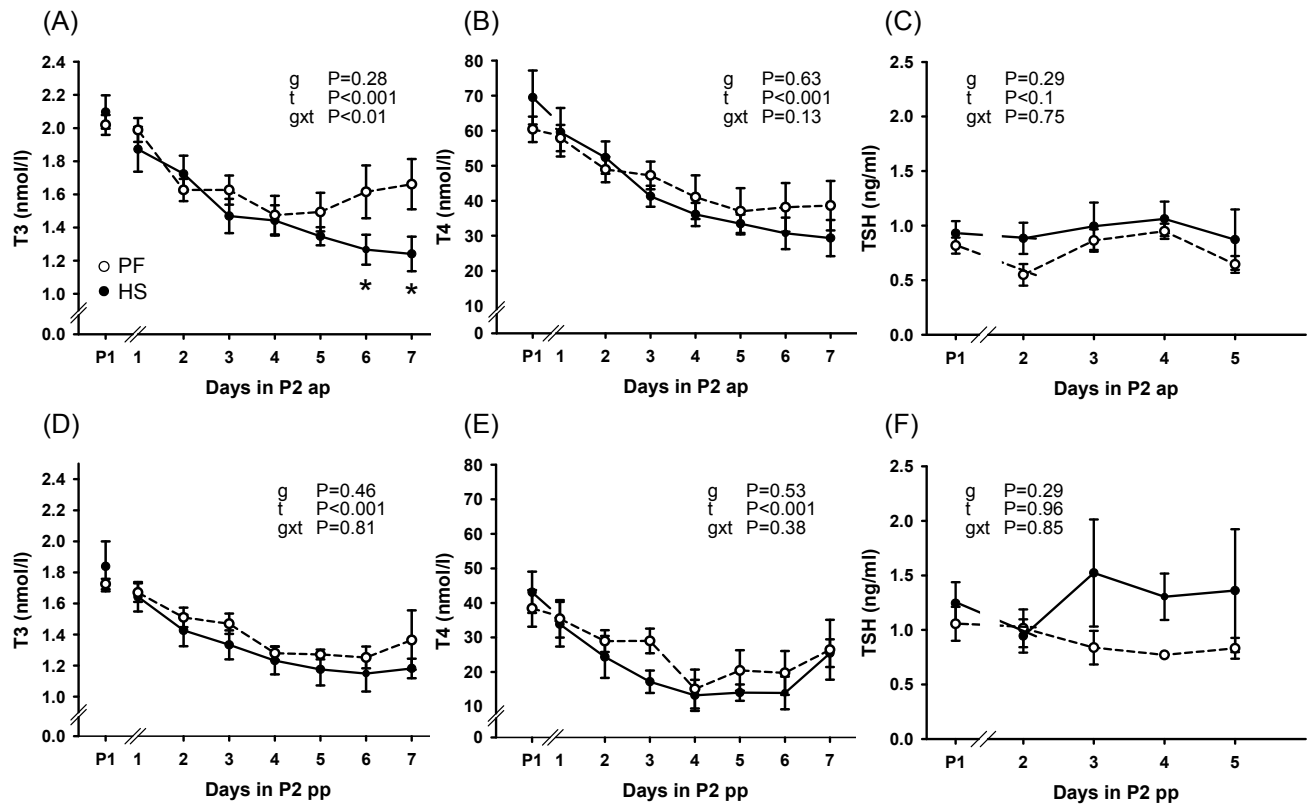

Supplement: Supporting Figure 2 [file joe-234-129-s002.pdf]
