# Supplementary material for: Hepatic thyroid signaling of heat-stressed late pregnant and early lactating cows
Source: J Endocrinol. 2017 May 12;234(2):129–41. doi: 10.1530/JOE-17-0066 (PMC5516449; doi:10.1530/JOE-17-0066)
Supplement: Supporting Table 1 [file erc-234-129-t001.pdf]

Supplementary Table 1. Liver proteins differentially expressed (P < 0.1) between thermoneutrality (P1) and after heat exposure (HS) during period P2 both in the ante-partum and post-partum stage identified by MALDI-TOF, MALDI-TOF/TOF and subsequent database search. Spot numbers refer to those proteins shown in Table 1 and Supplementary Figure 1.

Gel spot-no.	Accession	Description	Mass	Iso-electric point	Peptide Score	Ions score	Sequence coverage (%)	Sequence coverage (% ms ms)	Sequence coverage (% ms ms sig)	No.of pep-tides	Pep-tides with ions score	Pep-tides above identity thresh-old	Peptides above homology thresh-old	Rms error
**ante partum**														
588	114051576	arginase-1	35009	6.51	104	0	57.76	0	0	17	0	0	0	46.5
524	440911672	isovaleryl-CoA dehydrogenase	46698	7.21	123	0	49.3	5.37	0	20	0	0	0	45.76
782	548463703	3-hydroxyisobutyrate dehydrogenase	33462	7.18	91.1	0	54.09	0	0	17	0	0	0	13.49
492	440911294	glycine amidinotransferase	45945	7.26	93.6	21.1	45.14	4.24	0	16	1	0	0	31.55
528	155371831	beta-ureidopropionase	42816	6.89	88.5	0	52.86	0	0	20	0	0	0	44.11
786	440912826	proteasome subunit alpha	29583	6.6	99.4	0	58.94	4.18	0	17	0	0	0	21.09
628	300798377	coproporphyrinogen-III oxidase	50381	8.43	101	0	54.34	0	0	21	0	0	0	13.63
995	594629497	ferritin light chain	20078	5.84	84.8	0	69.71	0	0	12	0	0	0	35.34
1339	114051782	4-trimethylaminobutyraldehyde dehydrogenase	53977	5.88	89.2	27.7	44.33	3.04	0	17	1	0	0	40.87
423	402891049	UTP--glucose-1-phosphate uridylyltransferase	57831	8.39	152	40.6	37.33	2.71	2.71	20	1	0	1	23.45
639	150247075	galactokinase	42285	5.93	133	9.8	61.48	5.1	5.1	19	1	0	1	33.19
736	150247075	galactokinase	42285	5.93	79.2	0	43.88	4.85	0	15	0	0	0	19.86
241	27807355	NADH-ubiquinone oxidoreductase, complex1	79442	6.03	80.8	10.5	30.81	2.61	2.61	19	1	0	1	31.22
1345	556755352	adenosine kinase	33197	7	148	42.2	43.77	10.1	0	17	1	0	0	48.78
768	77735973	carbonyl reductase 1	30533	8.38	91.9	0	64.26	0	0	14	0	0	0	66.17
377	229299	catalase	57472	6.8	136	10.2	41.98	2.97	2.97	24	1	0	1	39.84
1350	229299	catalase	57472	6.8	94.1	0	36.24	0	0	18	0	0	0	41.3
574	229299	catalase	57472	6.8	105	0	39.6	0	0	18	0	0	0	56.19

Gel spot-no.	Accession	Description	Mass	Iso-electric point	Peptide Score	Ions score	Sequence coverage (%)	Sequence coverage (% ms ms)	Sequence coverage (% ms ms sig)	No.of pep-tides	Pep-tides with ions score	Pep-tides above identity thresh-old	Peptides above homology thresh-old	Rms error
**post-partum**														
199	78369436	3-ketoacyl-CoA thiolase	42131	8.01	166	28.2	65.99	6.55	6.55	22	1	0	1	47.23
207	440913558	3-ketoacyl-CoA thiolase	41546	7.76	210	21.2	74.74	9.95	0	27	1	0	0	53.4
327	741972663	3-ketoacyl-CoA thiolase	37646	7.5	104	0	61.02	0	0	17	0	0	0	20.12
1004	109659291	acetyl-Coenzyme A acetyltransferase 2	41170	6.94	103	17.1	52.14	4.03	4.03	18	1	0	1	18.3
1005	109659291	acetyl-Coenzyme A acetyltransferase 2	41170	6.94	104	7.5	51.89	6.8	0	19	1	0	0	39.89
1022	78365297	glycerol-3-phosphate dehydrogenase	37690	6.89	141	0	64.18	0	0	21	0	0	0	47.59
183	528995145	serine hydroxymethyltransferase	52969	8.11	140	0	47.73	0	0	24	0	0	0	56.97
170	41386780	UTP-glucose-1-phosphate uridylyltransferase	56903	8.09	148	0	54.72	0	0	24	0	0	0	51.09
191	640810185	pyruvate carboxylase	55792	8.59	103	0	46.73	0	0	20	0	0	0	72
194	741953862	fumarate hydratase	46426	7.77	201	93.4	49.54	5.3	5.3	20	1	1	1	53.86
315	77735973	carbonyl reductase 1	30533	8.38	109	0	68.95	0	0	16	0	0	0	20.03
862	594069864	regucalcin isoform X1	33590	5.49	135	28.1	55.15	12.62	0	19	2	0	0	37.73
893	742156827	sorbitol dehydrogenase	38099	7.27	183	42.7	62.36	12.08	0	21	2	0	0	40.32
663	62751593	profilin-1	15057	8.48	226	122.5	75.71	22.86	22.86	16	2	1	2	44.61
937	729433	protein disulfide-isomerase A3	56930	6.61	285	120.6	51.49	7.33	7.33	25	3	1	1	23.43
380	3219774	peroxiredoxin-6	24871	5.79	87	20.6	42.86	9.38	9.38	10	1	0	1	29.55
400	27806083	thioredoxin-dependent peroxide reductase	28195	7.67	117	17.4	50.58	7	5.45	15	1	0	1	52.42
890	29135275	thiosulfate sulfurtransferase	33296	7.24	152	50.8	48.48	11.78	6.4	14	1	0	1	54.37
934	78369302	catalase	59915	7.3	81.6	0	36.62	0	0	17	0	0	0	17.43
1066	77735641	persulfide dioxygenase ETHE1	27900	6.72	99.4	21.9	72.05	8.27	0	14	1	0	0	34.43
