# Supplementary material for: Hepatic thyroid signaling of heat-stressed late pregnant and early lactating cows
Source: J Endocrinol. 2017 May 12;234(2):129–41. doi: 10.1530/JOE-17-0066 (PMC5516449; doi:10.1530/JOE-17-0066)
Supplement: Supporting Table 2 [file erc-234-129-t002.pdf]

Supplementary Table 2. Primer sequences and annotations

Gene		Primer sequence (5’ to 3’)	Sequence ID	Product Size (base pairs)	Annealing (°C)	Efficiency (%)
DIO1	forward	aag tgc tcc tga cgc tgt tt	GI:169791015	158	60	91
	reverse	cca acg gac ctt cag gat aa			60	
THRA	forward	caa gtc act ctc tgc ctt taa cct	GI:114052505	220	60	92
	reverse	act ctg cac ttc tct ctc ctt cat			60	
NRF1	forward	aac gtc ctc atg tgt ttg aat cta	GI:402693415	207	60	89
	reverse	tct tgt act tac gca cca cat tct			60	
PPARGC1A	forward	gag ctt ctc aag tat ctg acc aca	GI:70778916	236	60	89
	reverse	tcc aca ctt aag gtt cgt tca ata			60	
RPS18	forward	ctt aaa cag aca gaa gga cgt gaa	GI:74268022	218	60	96
	reverse	cca cac att att tct tct tgg aca			60	
